# Integrin α2β1 decelerates proliferation, but promotes survival and invasion of prostate cancer cells

**DOI:** 10.18632/oncotarget.25945

**Published:** 2018-08-21

**Authors:** Marjaana Ojalill, Marjaana Parikainen, Pekka Rappu, Elina Aalto, Johanna Jokinen, Noora Virtanen, Elina Siljamäki, Jyrki Heino

**Affiliations:** ^1^ Department of Biochemistry, University of Turku, Turku, Finland

**Keywords:** prostate cancer, cancer stem cells, integrin, cell adhesion, p38

## Abstract

High expression level of integrin α2β1 is a hallmark of prostate cancer stem cell like cells. The role of this collagen receptor is controversial since it is down regulated in poorly differentiated carcinomas, but concomitantly proposed to promote metastasis. Here, we show that docetaxel resistant DU145 prostate cancer cells express high levels of α2β1 and that α2β1^High^ subpopulation of DU145 cells proliferates slower than the cells representing α2β1^Low^ subpopulation. To further study this initial observation we used Crispr/Cas9 technology to create an α2β1 negative DU145 cell line. Furthermore, we performed rescue experiment by transfecting α2 knockout cells with vector carrying α2 cDNA or with an empty vector for appropriate control. When these two cell lines were compared, α2β1 positive cells proliferated slower, were more resistant to docetaxel and also migrated more effectively on collagen and invaded faster through matrigel or collagen. Integrin α2β1 was demonstrated to be a positive regulator of p38 MAPK phosphorylation and a selective p38 inhibitor (SB203580) promoted proliferation and inhibited invasion. Effects of α2β1 integrin on the global gene expression pattern of DU145 cells in spheroid cultures were studied by RNA sequencing. Integrin α2β1 was shown to regulate several cancer progression related genes, most notably matrix metalloproteinase-1 (MMP-1), a recognized invasion promoting protein. To conclude, the fact that α2β1 decelerates cell proliferation may explain the dominance of α2β1 negative/low cells in primary sites of poorly differentiated carcinomas, while the critical role of α2β1 integrin in invasion stresses the importance of this adhesion receptor in cancer dissemination.

## INTRODUCTION

Integrin α2β1 is an abundant collagen receptor expressed mainly on epithelial cells and platelets. Additionally, many mesenchymal cells are α2β1 positive. This collagen receptor shows higher avidity to fibril forming collagens than to e.g. basement membrane collagen IV [1]. Integrin α2β1 also has a large number of other extracellular matrix (ECM) related ligands, such as tenascin C [2], laminins [3], proteoglycans endorepellin/perlecan [4] and decorin [5]. Experiments with α2 deficient mice have shown that this receptor is required in e.g. immune response, angiogenesis and platelet function [6–10].

In prostate cancer the role of α2β1 has remained unclear, despite the fact that the prostate stem cells, and accordingly the prostate cancer stem cell like cells, have been described to be α2β1^High^, CD44^High^, Trop2^High^, CD133^+^ and integrin α6^+^ cells [11–13]. Paradoxically, the expression of α2β1 is down regulated during prostate cancer progression [14, 15], but many studies propose an important role for α2β1 in bone metastasis [16–18]. The interplay between androgen receptor and α2β1 integrin expression [19] may partially explain the controversy.

Integrin α2β1 mediated signaling can activate p38 [20, 21] and ERK mitogen activated protein kinases (MAPKs) [22], and the signaling has been reported to either promote [22, 23] or inhibit cell proliferation [24, 25]. The cellular functions of α2β1 may be dependent on the organization of the surrounding ECM, since there are observations that collagen fibrils and monomers activate distinct signaling events [24, 25]. Moreover, there is significant cross-talk between integrins and growth factor receptors [26]. In the case of α2β1 at least platelet derived growth factor receptor [27], epidermal growth factor receptor [28] and hepatocyte growth factor receptor (c-met) [29] modify integrin expression or functions. Inhibition of cell division by fibrillar collagen has also been linked to the activation of p27^KIP1^, an inhibitor of cyclin-dependent kinase 2 [24, 25].

To study the paradoxical role of α2β1 integrin in prostate cancer we used Crispr/Cas9 technology to create an α2 integrin negative DU145 cell line and cDNA transfections to rescue α2 integrin, and an appropriate control (α2 integrin negative) cell line. We tested the cells in a spheroid model to mimic 3-dimensional tissue conditions. Our observations indicate that α2 integrin is a negative regulator of prostate cancer cell proliferation, but that it concomitantly promotes survival, drug resistance and invasion. Furthermore, the data from the RNA sequencing indicate that α2 integrin regulates the expression of several cancer progression and invasion related genes. Thus, α2β1 integrin may play a critical role in the protection of the stem cell like cells and in the invasion and metastasis.

## RESULTS

### Docetaxel resistant DU145 cells express high levels of α2β1 integrin and the α2β1^High^ subpopulation of DU145 cells proliferates slower than the α2β1^Low^ subpopulation

**Figure 1:**
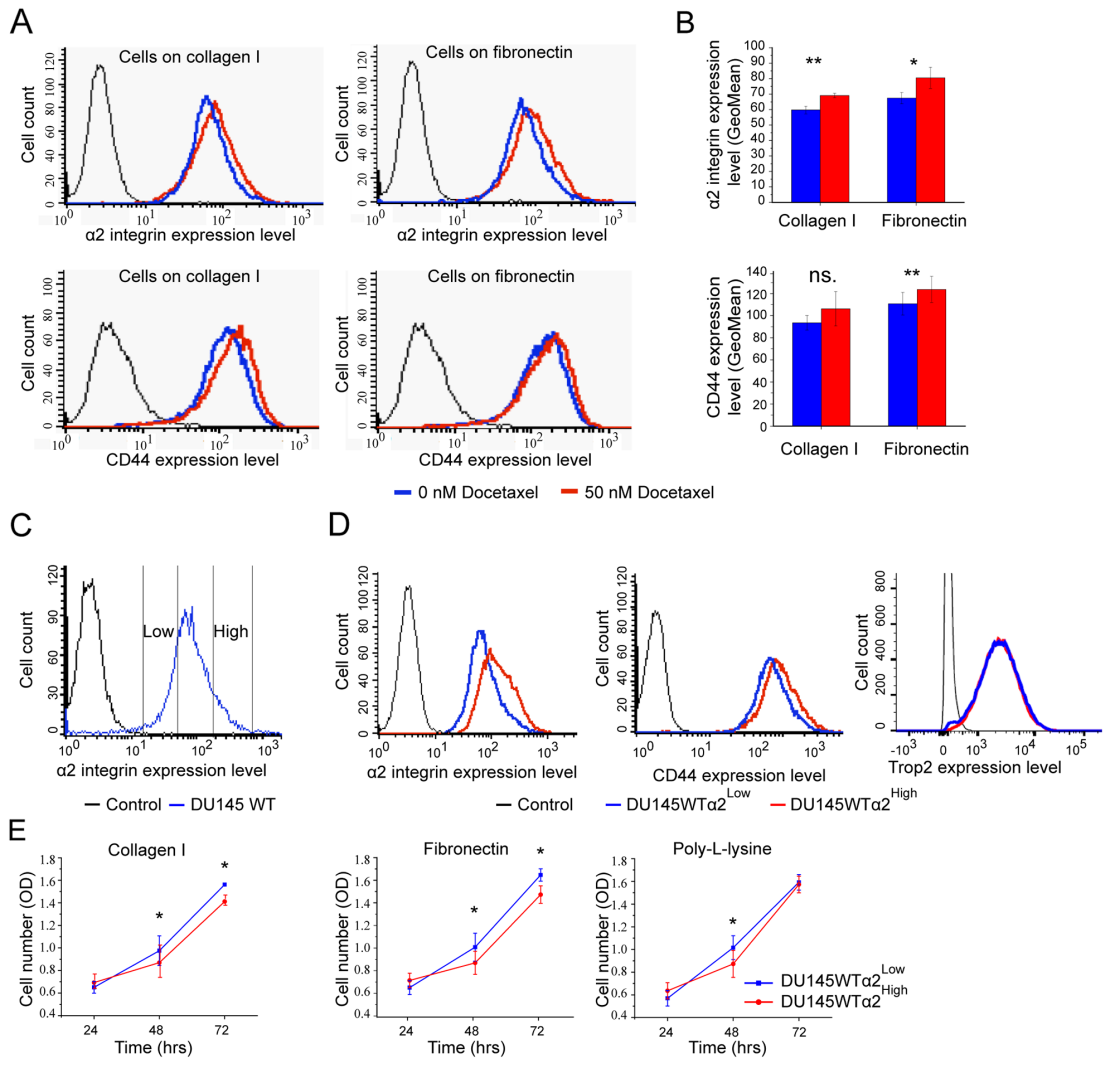
DU145 cells that survive docetaxel treatment show increased expression of stem cell markers: α2 integrin and CD44 **(A)** The representative FACS plots show increased surface expression levels of α2 integrin and CD44 on cells that have been on collagen I or on fibronectin (5 μg/cm^2^) coated plates and treated with 50 nM Docetaxel for 24 hours. **(B)** The quantification of α2 integrin and CD44 cell surface expression based on GeoMean values. Data as mean ± SEM (n=3). ^*^ = P < 0.05; ^**^ = P < 0.01; ns. = not significant. **(C)** The FACS plot illustrates the sorting of DU145WT cells into DU145WT (α2^High^) and DU145WT (α2^Low^) subpopulations based α2 integrin expression level on cells. **(D)** Representative FACS plots for α2 integrin, CD44 and Trop2 expression on DU145WT (α2^High^) and DU145WT (α2^Low^) subpopulations analyzed in the 5th passages after sorting. **(E)** Quantification of the proliferation of DU145WT (α2^High^) subpopulation compared to DU145WT (α2^Low^) subpopulation on collagen I, fibronectin and poly-l-lysine measured with the WST-8 assay. Data are mean absorbance values (OD) at 490 nm (n=3). ^*^ = P < 0.05.

DU145 is an α2β1 integrin positive prostate cancer cell line [19] originally derived from a central nervous system metastasis. In general, prostatic epithelial cells and prostate cancer cells are known to use α2β1 integrin in adhesion to various collagen subtypes and bone matrix [16, 17, 30]. We exposed DU145 cells to 50 nM docetaxel and measured their α2β1 and CD44 expression levels by flow cytometry before and after the treatment. The surviving cells expressed significantly higher α2β1 levels than untreated cells (Figure 1A, 1B). The result was the same when the cells were plated either on collagen I or fibronectin (Figure 1A, 1B). Docetaxel is known to block mitosis by inhibiting mitotic spindle assembly, accordingly here DU145 cells treated with 50 nM docetaxel were significantly converted from G1 to G2/M phase (Supplementary Figure 1A, 1B). The high α2β1 expression could be linked to survival also without ligand binding by α2β1 integrin. Similarly, the surviving cells expressed more CD44 (Figure 1A, 1B), suggesting that the cells with stem cell markers might be more drug resistant. The most probable reason for the decreased sensitivity was speculated to be the potentially lower proliferation rate of α2β1^High^/CD44^High^ cells.

To further test this hypothesis we selected DU145-α2β1^High^ and DU145-α2β1^Low^ subpopulations by using specific antibodies and sorting flow cytometry (Figure 1C) and examined their proliferation on collagen I, fibronectin and poly-l-lysin (Figure 1E). The results indicated that the number of α2β1^High^ cells increased slower that α2β1^Low^ cells and that the difference was not dependent on the binding of α2β1 to its ligand, collagen I. Interestingly, the cells selected based on their high α2β1 integrin expression levels also expressed high levels of CD44, but there was no difference in Trop2 expression levels (Figure 1D). Thus, we concluded that α2β1^High^/CD44^High^ DU145 cells proliferate slower than DU145 cells in general. However, these results only indicated an association between α2β1 integrin, survival and proliferation, and further experiments were needed to study the functions of α2β1 integrin in cell regulation.

### In a genetic model α2 integrin positive DU145 cells proliferate slower and are more resistant to docetaxel than α2 integrin negative cells

**Figure 2:**
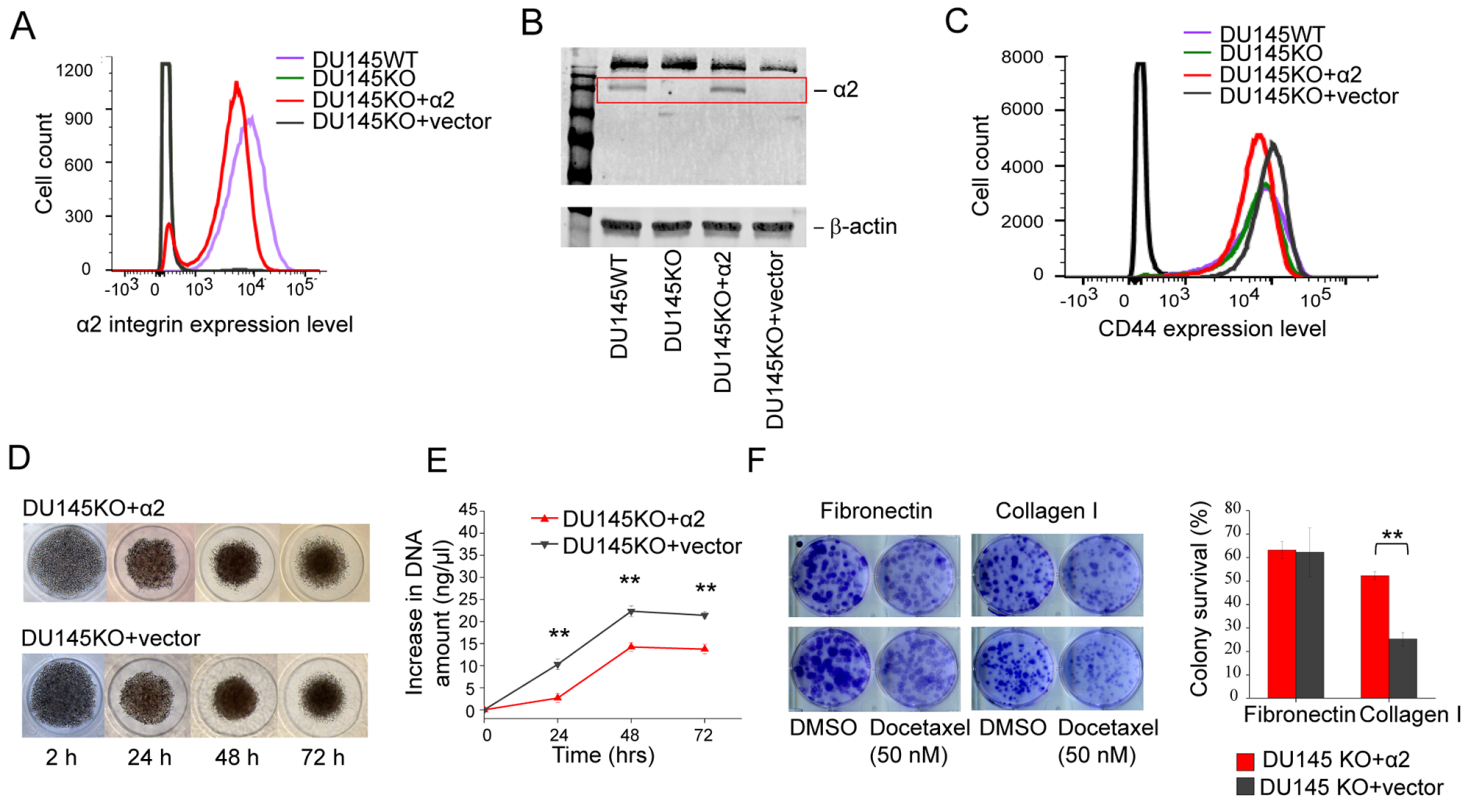
α2β1 integrin expression decelerates cell proliferation, but promotes resistance to docetaxel **(A)** FACS plot showing α2 integrin cell surface expression on DU145WT (purple), DU145KO (green), DU145KO+α2 (red) and DU145KO+vector (grey) cells. **(B)** Representative western blot demostrating α2 integrin expression in whole cell lysates of DU145WT, DU145KO, DU145KO+α2 and DU145KO+vector cells. **(C)** FACS plot showing surface expression levels of CD44. DU145WT (purple), DU145KO (green), DU145KO+α2 (red) and in DU145KO+vector (grey) cells. Expression of CD44 is independent from α2 and is not regulated by acquired expression of α2 integrin. **(D)** Representative images of DU145KO+α2 and DU145KO+vector cells grown as 3D spheroids up to 72 hours. **(E)** Quantification of DNA amount to measure the proliferation of DU145KO+α2 and DU145KO+vector cells in 3D spheroid culture. Data are mean ± SEM (n=3). ^**^ = P < 0.01. **(F)** Representative images of DU145KO+α2 and DU145KO+vector cells in colony formation and survival assay. Below the quantification of colony survival assay comparing DU145KO+α2 and DU145KO+vector cells on fibronectin and collagen I. Data are mean ± SEM (n=3). ^**^ = P < 0.01.

We successfully used Crispr/Cas9 technology to create α2 integrin negative DU145 cells (DU145KO). The realization of knockout was confirmed by sequencing the PCR product received in the amplification of genomic DNA in the area of interest (Supplementary Figure 2). We did not, however, consider DU145WT (wild type) versus DU145KO to be an optimal pair for comparisons for two reasons: i) DU145WT (wild type) cells were indicated to be a mixture of α2β1^High^ and α2β1^Low^ cells with distinct properties, and ii) complex transfection and selection procedure may have had unpredicted influences on the DU145KO cell line. Therefore we used cDNA transfections to create two more cells lines: DU145KO+α2 that has rescued expression of α2 integrin and an α2 negative vector control DU145KO+vector line (Figure 2A, 2B). The α2β1 expression levels were confirmed using specific antibodies in flow cytometry (Figure 2A) and western blotting (Figure 2B). Overexpression of α2 integrin had no effect on the CD44 expression level on cell surface (Figure 2C). Thus the two stem cell markers are not directly regulated by each other.

The proliferation of the two cell lines was tested in spheroids instead of monolayer cultures since α2β1 integrin specific signaling may be more apparent in 3D environment [1]. We tested the proliferation of α2 positive DU145KO+α2 and α2 negative DU145KO+vector cells by measuring the increase in the DNA content of the spheroids. The spheroids formed by α2 negative cells were slightly more compact and therefore had smaller diameter (Figure 2D), but the amount of DNA in these spheroids increased significantly faster indicating higher cell proliferation rate (Figure 2E). When the resistance to docetaxel was measured using a colony survival assay, α2β1 positive cells were significantly more resistant to this drug than their α2 negative counterparts on collagen, but not on fibronectin (Figure 2F).

### α2 positive DU145 cells are more invasive than α2 negative cells

**Figure 3:**
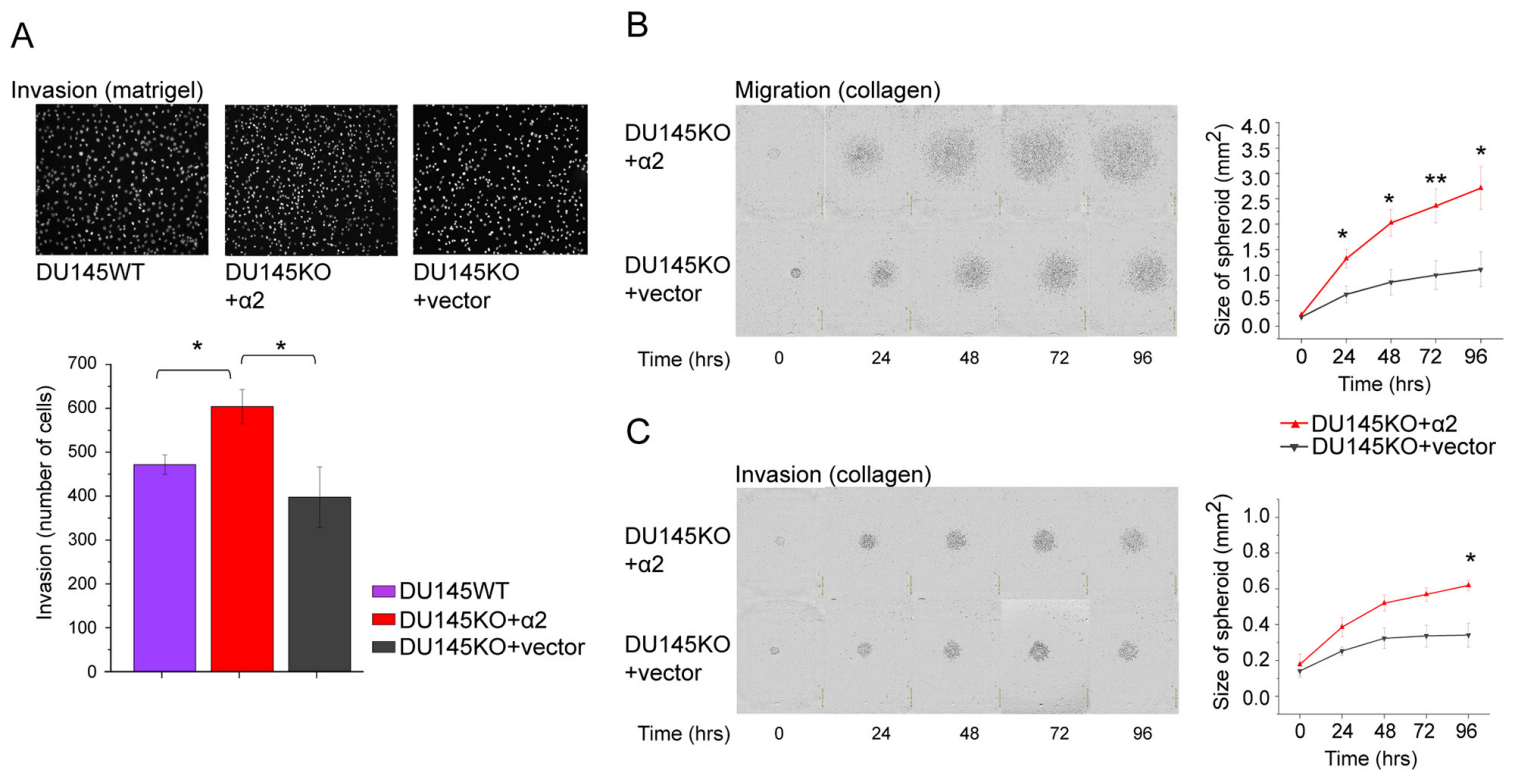
α2 integrin positive DU145 cells are more invasive than α2 negative cells **(A)** Representative microscopic images (10 x) of DU145WT, DU145KO+α2 and DU145KO+vector cells that have invaded through matrigel in the transwell invasion assay. Below, the quantification of the invaded cells. Mean ± SEM (n=5). ^*^ = P < 0.05. One way ANOVA and Tukey HSD post hoc test. **(B)** Representative images of DU145KO+α2 and DU145KO+vector cells in migration assay. Cells were allowed to migrate out of spheroids on the collagen I coated surface for 96 hours. The quantitation of cell migration as the area covered by DU145KO+α2 and DU145KO+vector cells. Data are mean ± SEM (n=4). ^*^ = P < 0.05; ^**^ = P < 0.01. **(C)** Representative images of DU145KO+α2 and DU145KO+vector cells in invasion assay. Cells were allowed to invade out of spheroids into the collagen I gel for 96 hours. The quantitation of cell invasion as the area covered with DU145KO+α2 and DU145KO+vector cells. Data are mean ± SEM (n=4). ^*^ = P < 0.05.

We speculated that the loose growth mode of α2 positive cells in spheroids may be due to increased cell mobility and therefore we established both cell migration and invasion assays. DU145KO+α2 cells showed significantly higher rate of invasion through matrigel in transwell assay when compared to DU145WT or DU145KO+vector cells (Figure 3A). We also tested the cells in another type of assay, in which 3 day spheroids were plated on collagen I (Figure 3B, migration assay) or embedded between collagen layers (Figure 3C, invasion assay) and the diameter of the area covered by cells was measured every 24 hours during 4 days. DU145KO+α2 cells migrated (Figure 3B) and invaded (Figure 3C) significantly faster than their α2 negative counterparts.

The difference in the migration rate between α2β1^High^ and α2β1^Low^ cells was also evident when another prostate cancer cell line, PC3 was tested. These cells differ from DU145 in many characteristics, e.g. PC3 α2β1^High^ cells have lower CD44 expression levels than PC3 α2β1^Low^ cells. At the same time the expression level of Trop2 is much higher in PC3 α2β1^High^ cells. In agreement with our DU145 cell results, PC3 cells that survive docetaxel treatment have high α2 integrin expression level. The results with PC3 cells are shown in Supplementary Figure 3.

### Activation of p38 MAPK mediates the effects of α2β1 on cell proliferation and invasion

**Figure 4:**
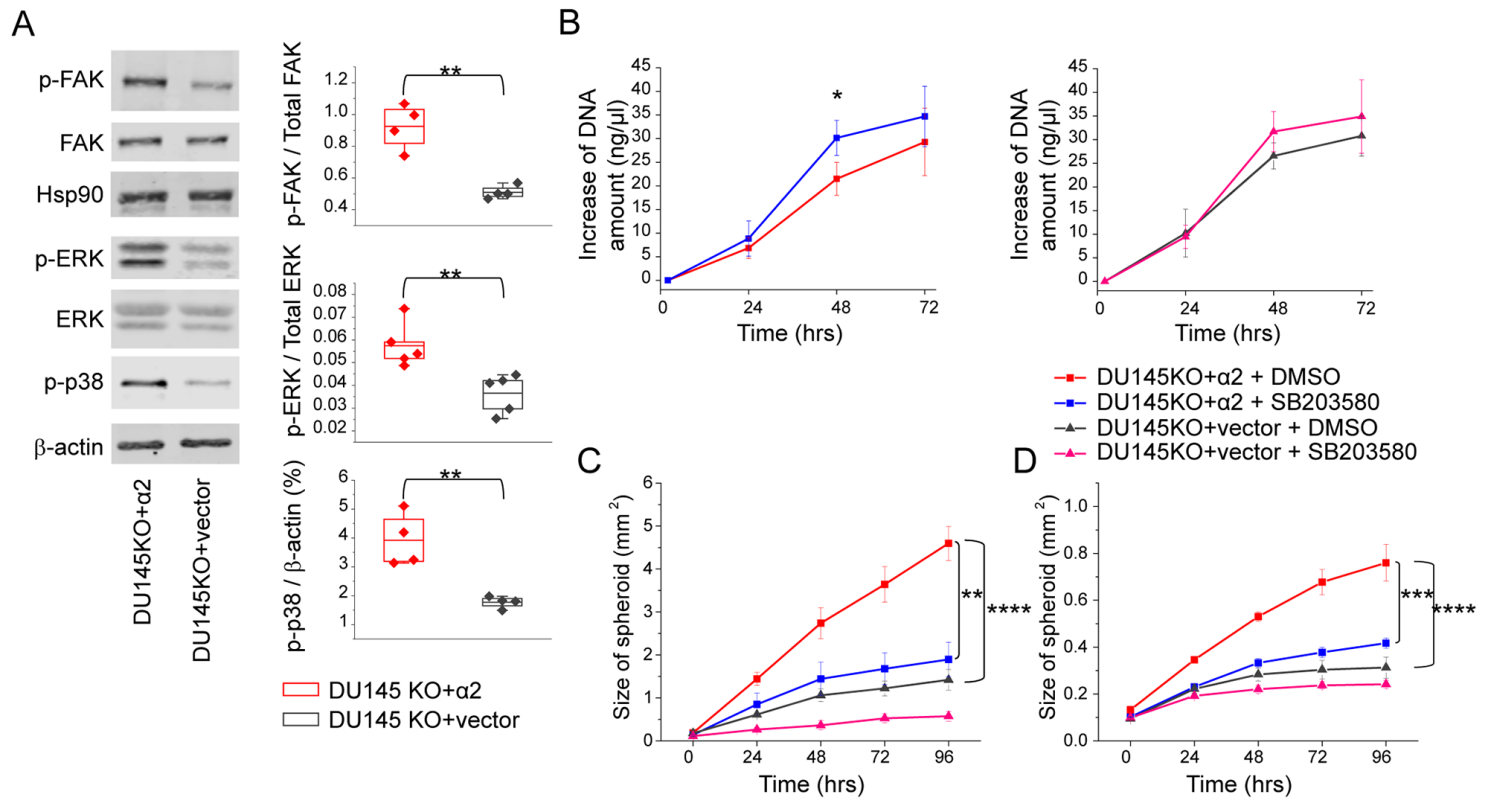
α2β1 integrin suppresses cell growth and promotes migration and invasion by increasing the phosphorylation of p38 MAPK **(A)** Representative western blot indicating that α2 integrin expression on DU145 prostate cancer cells increased phosphorylation of FAK, ERK and p38 MAPK proteins when cells were plated on collagen I coated surface. The quantification of the phosphorylated FAK/ total FAK and phosphorylated ERK/ total ERK is shown; phosphorylation of p38 MAPK is shown as % of β-actin (a loading control). Box plot shows data from 4 or 5 independent experiments (dots) and the mean from all experiments ±SEM. ^**^ = P < 0.01. Student’s *t*-test. **(B)** p38 MAPK inhibitor SB203580 treatment (10 μg/ml) increased proliferation of DU145 cells. Proliferation of DU145KO+α2 and DU145KO+vector cells was assessed based on the change in DNA amount in 3D spheroids. Mean (n = 3) ±SEM. ^*^ = P < 0.05. Student’s *t* test. **(C)** Inhibition of p38 MAPK with SB203580 (10μg/ml) results significantly decreased migration of DU145KO+α2 cells on collagen I. Mean (n = 3) ±SEM. ^**^ = P < 0.01, ^***^ = P < 0.001. One way ANOVA and Tukey HSD post hoc test. **(D)** Invasion capability of DU145KO+α2 cells into collagen gel decreased significantly when cells were treated with p38 MAPK inhibitor SB203580 (10μg/ml). Mean (n = 3) ±SEM. ^***^ = P < 0.001. One way ANOVA and Tukey HSD post hoc test.

In many cell types, integrin α2β1 signaling is known to induce the phosphorylation of focal adhesion kinase (FAK) [31] and activate growth promoting ERK MAP-kinase [21]. Integrin α2β1 has also frequently been linked to the phosphorylation of p38 MAPK [20, 21]. Here, we plated α2 negative DU145KO+vector cells and α2 positive DU145KO+ α2 cells on collagen and measured the activation of FAK, ERK and p38 by western blotting. The phosphorylation of all three proteins was clearly more prominent in α2 positive cells (Figure 4A).

Next we tested the effect of selective p38 inhibitor SB203580 on cell proliferation, invasion and migration. The inhibition of p38 by SB203580 was confirmed by measuring the phosphorylation of downstream signaling protein CREB (cAMP response element-binding protein). In DU145WT and DU145KO+α2 cells the treatment with SB203580 (10 μg/ml) resulted in 40-50% reduction of CREB activation (Supplementary Figure 4A, 4B). SB203580 increased significantly the proliferation (the amount of DNA) in spheroid cultures of DU145KO+α2 cells at 48 h time point (Figure 4B). It also slightly enhanced the proliferation of α2 negative cells, but the increase was not statistically significant (Figure 4B).

In the migration and invasion assays SB203580 was a potent inhibitor of DU145KO+α2 cells (Figure 4C, 4D). In the presence of the p38 inhibitor the migration and invasion by these cells were reduced to the same level as was measured with their α2 negative counterparts (Figure 4C, 4D).

Thus, we conclude that the effects of α2β1 expression on proliferation, migration and invasion by prostate cancer cells may be at least partially due to the elevated p38 phosphorylation.

### Integrin α2β1 regulates cancer progression related genes

**Figure 5:**
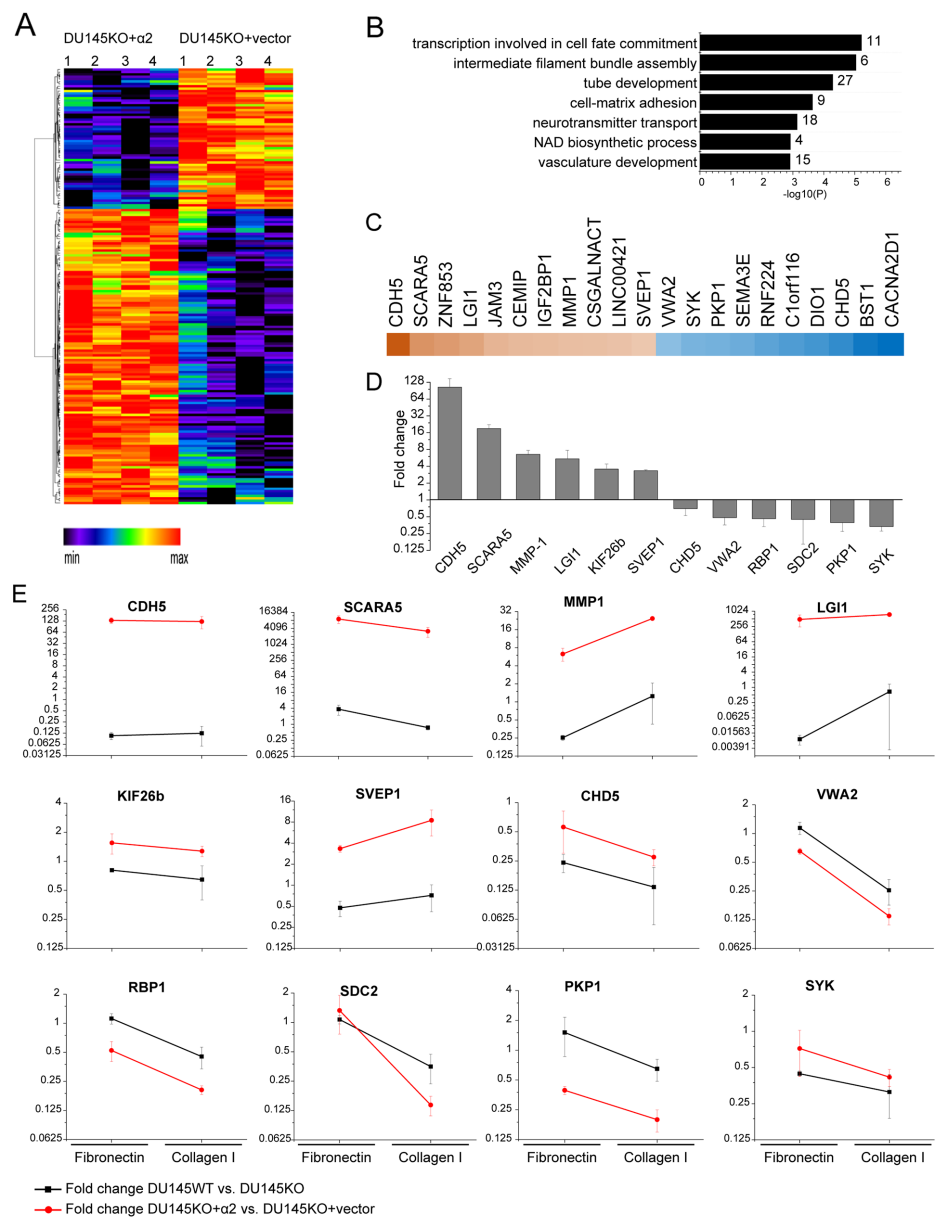
α2β1 integrin regulates the expression of cancer associated genes **(A)** Differential gene expression pattern of DU145KO+α2 compared to DU145KO+vector cells. Hierarchical clustering of differentially expressed (DE) genes based on relative gene expression levels detected in RNA sequencing. Red and black colors represent over and under expressed genes, respectively. **(B)** Differential gene expression in DU145KO+α2 compared to DU145KO+vector cells has an effect on several biological processes. Significantly over-represented gene ontology terms based on Metascape analysis. **(C)** Heatmap of the most up and down regulated genes in DU145KO+α2 cells when compared to DU145KO+vector cells. Brown and blue colors represent higher and lower expression levels, respectively. **(D)** Real time PCR analysis of selected, α2β1 integrin associated genes, in DU145KO+α2 and in DU145KO+vector cells grown as 3D spheroids. Difference is shown as the fold change of relative mRNA expression level in DU145KO+α2 compared to DU145KO+vector expression level. Data are mean (n = 3) ±SEM. **(E)** Effect of cell adhesion to extracellular matrix on the selected α2 integrin associated genes. Cells were grown either on collagen I or on fibronectin for 72h, after that relative mRNA levels of selected genes was analyzed by qPCR. Each data point represents the ratio of mRNA levels in α2 positive / α2 negative cells. Both, DU145KO+α2 / DU145KO+vector (red line) and DU145WT / DU145KO (black line) comparisons were made. Data are mean (n = 3) ±SEM.

We used RNA sequencing to analyze the putative differences in the gene expression pattern of α2 negative DU145KO+vector cells and α2 positive DU145KO+α2 cells (Figure 5). For that purpose we isolated RNA from cells grown in spheroid cultures. The analyses unveiled several differences (Figure 5A). The top seven overrepresented biological process gene ontology terms among the DE genes from Metascape analysis at http://metascape.org [32] are shown in Figure 5B. Figure 5C shows top ten genes with the most significant increases or decreases. For further experiments we selected 12 genes based on three criteria: i) difference in expression (up or down regulated) when α2 negative DU145KO+vector cells and α2 positive DU145KO+α2 cells were compared, ii) similar difference in expression when α2 negative DU145KO cells and α2 positive DU145KO+α2 cell were compared, and iii) preferentially previously described connection to cancer progression. The up regulated genes included: cadherin 5 (CDH5), scavenger receptor class A member 5 (SCARA5), matrix metalloproteinase 1 (MMP1), leucine rich glioma inactivated 1 (LGI1), kinesin family member 26b (KIF26b) and sushi, von Willebrand factor type A, EGF and pentraxin domain containing 1 (SVEP1). The down regulated genes included: chromodomain-helicase-DNA-binding protein 5 (CHD5), von Willebrand factor A domain containing 2 (VWA2), retinol binding protein 1 (RBP1), syndecan 2 (SDC2), plakophilin 1 (PKP1) and spleen associated tyrosine kinase (SYK). The differential expression between α2 positive and α2 negative cells was confirmed by quantitative real time PCR (Figure 5D).

Since MMP1 was remarkably upregulated in α2 positive cells and these cells showed increased mobility, we also tested the effects of a broad spectrum MMP inhibitor, N-Isobutyl-N-(4-methoxyphenylsulfonyl)glycyl hydroxamic acid (NNGH), in migration and invasion assays. The migration of DU145KO+α2 and DU145WT was significantly reduced in the presence of 1 μM NNGH. The invasion was not, however, influenced at the concentration used (Supplementary Figure 5). The results confirmed that MMPs are involved in the mobility of DU145 cells.

Despite the fact that the listed changes in gene expression could be linked to the presence of α2β1 integrin, the result did not answer the question, whether these genes are also directly regulated by α2β1 binding to its ligand. Therefore, we plated the cells either on collagen I or on fibronectin and analyzed the mRNA levels by qPCR. Ratio of [mRNA level in α2^+^ cells] / [mRNA level in α2^-^ cells] was calculated on both matrices (Figure 5E). The experiment was also repeated using DU145WT (α2 positive) and DU145KO cells (α2 negative). The expression of maximally seven out of twelve genes (MMP-1, SVEP1, SYK, VWA2, PKP1, RBP1, SDC2) changed after α2β1–collagen interaction in the same manner (up or down regulation) than in α2 expressing cells, which leaves open the possibility that α2β1 may also affect the expression of some genes in a mechanism that is independent of direct ligand binding.

The majority of the twelve selected, α2β1 integrin associated genes were regulated in a similar manner when PC3 cells and DU145 cells were compared. When their gene expression patterns were compared after plating on collagen I and fibronectin nine out of twelve genes showed up/down regulation in both cells lines, whereas three genes (SCARA5, SVEP1, SDC2) were differentially regulated (Supplementary Figure 3).

To study the role of p38 activation in the α2β1 integrin associated changes in gene regulation, we plated DU145KO+α2 cells on collagen or fibronectin in the presence of SB203580 or DMSO (negative control) and measured mRNA levels (at 72 h) by real time PCR. In the case of five out of six genes that were up regulated in the presence of α2β1 integrin (MMP-1, SVEP1, SCARA5, CDH5 and KIF26b), SB203580 reduced mRNA levels below 50% of control on both collagen (MMP-1 35%, SVEP1 37%, SCARA5 40%, CDH5 43% and KIF26b 21% of control) and fibronectin (MMP-1 38%, SVEP1 42%, SCARA5 16%, CDH5 46% and KIF26b 32% of control). The differences were smaller or not constant in the other seven selected genes. Thus, in most cases the genes, which were positively regulated in the presence of α2β1 integrin, were also dependent on active p38, whereas no similar association could be seen in genes that were down regulated in the presence of α2β1 integrin.

## DISCUSSION

The stem cell theory of cancer suggests that in a tumor very few cells can sustain the malignant process. The idea has important implications: firstly, the cancer stem cell may be the only cell type that gives rise to metastases, and secondly, if anti-cancer therapies are not able to kill the cancer stem cells, the tumor will soon grow back. In prostate cancer, high α2β1 integrin expression is supposed to be one of the hallmark signs of the stem cell phenotype [11]. During the progression of the disease α2β1 positive cells are getting more and more rare [14, 15], but still the collagen receptor may play an important role in metastasis [16–18]. Our experiments were planned to solve this controversy and unveil the function of α2β1 integrin in prostate cancer cells at molecular level.

Using DU145 cell line we showed that the population of cells, which survived the docetaxel treatment, expressed slightly more α2β1 integrin than non-treated DU145 cells. Also previous studies have shown that chemoradiotherapy resistant DU145 cells have elevated levels of two other stem cell markers, namely CD44 and CD133 [33]. Furthermore, CD44 and α2β1 positive DU145 cells are more tumorigenic than the cells that have lower expression levels of these marker proteins [34]. Here, we used flow cytometry to separate α2β1^High^ and α2β1^Low^ DU145 cell subpopulations and demonstrated the slower proliferation rate of the α2β1^High^ population. Increased survival during paclitaxel treatment and slow proliferation may be linked to each other since paclitaxel affects cell division and kills actively growing cells. Previously, up regulation of phosphatidyl inositol-3-kinase/Akt and MAPK pathways have been linked to the resistance of microtubule-targeting drugs in prostate cancer [35]. Interestingly, both pathways can also be regulated by α2β1 integrin [22, 36].

The observations based on the comparison of α2β1^High^ and α2β1^Low^ subpopulations demonstrated a link between the collagen receptor expression and the stem cell like phenotype. However, it remained still unknown, whether α2β1 directly participates in the process that dictates the stem cell like behavior. Therefore, we used Crispr/Cas9 technology to knockout α2 integrin in DU145 cells. To create two cell lines that could be compared to each other in an appropriate manner, we transfected DU145KO cells either with α2 integrin cDNA or with empty vector. We choose to study the cellular functions in spheroids instead of monolayer cultures since pronounced α2β1 integrin signaling may be dependent on the 3D environment [1]. Our results indicated that α2β1 integrin decelerates prostate cancer cell proliferation. Importantly, this observation may explain the decrease in the number of α2β1 positive cells during prostate cancer progression. Still α2β1 positive cells migrated and invaded significantly more effectively than their α2β1 negative counterparts. Thus the presence of this receptor may give remarkable advantage in cancer dissemination related processes. This may explain the presence of α2β1 integrin in metastatic prostate cancer cells and stress the role of α2β1 positive cells especially in the early dissemination.

Our data also indicated that α2β1 integrin regulates several cancer associated genes. A large increase was seen in cadherin 5 (CDH5, vascular endothelial cadherin) that plays an important role in homotypic cell–cell adhesion among epithelial cells. CDH5 is a marker of poor survival in human gastric cancer [37]. Seemingly paradoxically, α2β1 expression also increased the mRNA levels of two tumor suppressors, namely SCARA5 (Scavenger receptor class A, member 5) and LGI1 (Leucine-rich, glioma inactivated 1). The up regulation of these anti proliferation genes is, however, in accordance with the fact that α2β1 integrin expression also suppressed cell proliferation. Concomitantly α2β1 down regulated the expression of two other tumor suppressors, namely PKP1 (plakophilin 1) and CDH5 (chromodomain helicase DNA binding protein 5). Largest down regulation was seen in syndecan 2 expression. Interestingly, syndecan 2 is strongly up regulated in prostate cancers with high Gleason score [38]. In Gleason 3-5 prostate cancers α2β1 integrin is significantly decreased [15], suggesting that the expression levels of α2β1 and syndecan 2 may be linked *in vivo*, too.

In prostate cancer cells, MMP-1 may be a critical, invasion promoting factor [39]. Here, in accordance with the observation that α2β1 promotes cell invasion, it remarkably up regulated the expression of MMP-1. Our previous studies have also indicated the association of α2β1 integrin and MMP-1 expression in other cell types [40].

In maximally 7 out of 12 cases the genes that were up or down regulated in α2β1 positive cells, when compared to their α2β1 negative counterparts, were also regulated after α2β1–collagen interaction. However, in many experiments α2β1 related effect was similar when cells were plated either on collagen or fibronectin. Thus, we cannot exclude the possibility that α2β1 may also have ligand independent functions. Ligand independent integrin signaling has been shown to occur in some experimental models [41], but in general these mechanisms are poorly known.

We also suggest that the activation of p38 MAPK by α2β1 integrin is one of the major pathways suppressing proliferation and activating invasion. Previous studies have shown that p38 often acts as a protumorigenic factor [42, 43]. Based on histological staining of human prostate tumors p38 MAPK expression has a significant, positive correlation with carcinogenesis, cancer progression and patient survival [44]. Regulation of cell locomotion by p38 has been documented in many cell types [45], including DU145 cells [46]. Here, MMP-1 expression was dependent on p38 activity, which further stresses the role of this signaling pathway in the α2β1 integrin dependent invasion.

To conclude, our results suggest that α2β1^High^ cells have stem cell like properties and these cells may play an important role in the sustenance of prostate cancer and in the processes leading to dissemination. Thus, this cell type should be the primary target when new therapies are developed.

## MATERIALS AND METHODS

### Cell lines

The human prostate cancer cell-lines DU145 and PC3 were obtained directly from ATCC. DU145WT and PC3 cells have endogenous expression of α2 integrin. To create an α2 negative cell line genome editing method was applied by transfecting DU145 cells with all-in-one Crispr/Cas9 vector (Sigma, HS0000253951 gRNA sequence: GTTACTGGTTGGTTCACCCTGG), transfected cells were selected based on the expression of GFP, allowed to grow and checked for α2 integrin surface expression. The cells that had been transfected with Crispr/Cas9 vector and successful ITGA2 knockout had taken place, were named as DU145KO. A smaller population of cells with Crispr/Cas9 vector, but still α2 integrin expression, was sorted out by flow cytometer. DU145KO cells were stably transfected either with pAWneo2 vector carrying α2 integrin cDNA [47] to regenerate α2 positive cell line (DU145KO+α2 cells) or with empty pAWneo2 vector to create a proper α2 negative control cell line (DU145KO+vector). DU145KO+α2 and DU145KO+vector cells were grown in the presence of 0.25 mg/ml geneticin (GIBCO). All cells were maintained in RPMI1640 medium (Lonza), supplemented with 10% fetal calf serum (FCS, Biowest), 2 mM Ultraglutamate, 100 U/ml penicillin and streptomycin (Lonza). In the indicated experiments also serum-free keratinocyte medium (KSF) with 5 ng/ml human recombinant EGF, 50 μg/ml bovine pituitary extract (all from Gibco), supplemented with 2 ng/ml recombinant human leukemia inhibitory factor and 2 ng/ml stem cell factor (Sigma Aldrich) was used. Cells were routinely screened with MycoAlert™ PLUS *Mycoplasma* detection kit (Lonza).

### Spheroid cultures

Spheroids were made in micro-molds according to the manufacturer’s instructions (3D Petri Dish, MicroTissues) with 2.8 × 10^5^ cells in one mold (8000 cells in one spheroid). Spheroids were grown in RPMI 1640 serum-free (SF) medium.

### RNA extraction

After 72 hours, total RNA was extracted from the cells cultured in spheroids by using NucleoSpin RNA (Macherey-Nagel) kit according to the manufacturer’s instructions. The quality and quantity of the RNA was checked using Nanodrop ND-2000 spectrophotometer (Thermo Scientific).

### RNA sequencing

The samples were prepared for the sequencing using Illumina TruSeq Stranded mRNA Sample Preparation Kit and 0.9 μg of high-quality RNA from each sample was analyzed with HiSeq^®^ 2500 Sequencing System (Illumina) using single-end sequencing chemistry and 50bp read length. Total of 4 samples 4 independent biological replicates were analyzed and run in two lanes. The reads obtained from the instrument were base called using the instrument manufacturer’s Bcl2fastq version 1.8.4 base calling software. The read alignment was performed in two stages: first, the reads were aligned against the reference genome (human hg38, downloaded from Illumina iGenomes web site) using TopHat version 2.1.0 [48]. Then the reads were associated with known genes and the number of reads associated with each gene was counted using subreads package (v. 1.5.0) [49]. Only uniquely aligned reads were used for the downstream analysis.

The gene-wise read counts were normalized using the TMM normalization method of the edgeR R/Bioconductor package. For statistical testing the data were further transformed using the voom approach in the limma package. R package Limma [50] was used for performing the statistical testing between the groups. The gene was determined as differentially expressed (DE) if the fold change was > 2 between comparison groups. The DE genes were ranked as follows: first the genes inside each comparison were ranked independently based on p-values (increasing order) and absolute fold changes (decreasing order). Next the average ranks for these two were calculated. As these values do not follow the original value range, these average ranks were further ranked again to get the ranks from 1 to the number of genes, rank of 1 meaning the highest possible rank (most differentially expressed gene based both on p-value and fold change). In tie situations, the order was randomized.

The sequencing data have been deposited in NCBI's Gene Expression Omnibus and are accessible through GEO Series accession number GSE111507 (https://www.ncbi.nlm.nih.gov/geo/query/acc.cgi?acc=GSE111507).

### Matrigel transwell invasion assay

The ability of DU145 and PC3 cells to invade through Matrigel was examined using Matrigel coated invasion chambers (#354480, Corning) according to the manufacturer’s instructions. Samples were prepared in triplicates, 1 × 10^5^ cells were seeded into the top chamber of 24-well insert in 0.5 ml 0.1 % BSA-containing RPMI SF media. Medium with 10 % FCS was used as a chemoattractant in the bottom chamber. After 24 hours, residual cells on the top surface of insert were removed, membranes were fixed with cold methanol and nuclei were stained with DAPI. Nuclei were imaged from 9 fields of each membrane, and counted with ImageJ. Average nuclei count for each cell type was obtained, normality of the data was tested with the Shapiro-Wilk test and statistical significances were obtained by comparing the average nuclei counts with one-way ANOVA test and pairwise comparisons were made with the Tukey HSD post hoc test.

### Out of spheroid cell migration on and invasion through collagen I

The role of α2 integrin in the DU145 cell migration on collagen I or invasion through 3D collagen I gel was studied by placing spheroids as triplicates on collagen I coated 96-well plates. When invasion was tested a collagen I gel (2.0 mg/ml bovine skin collagen I, Nutragen, Advanced BioMatrix; 4 mM Tris-HCl, pH 7.3, and 20 mM NaOH in serum-free RPMI) was layered on the top of the spheroids. KSF medium (described above) was placed above the collagen gel. Spheroids were allowed to migrate or invade for 96 hours and imaged every 24 hours with IncuCyte ZOOM System (Essen Bioscience). ImageJ was used to detect the area covered with cells. Mean cell covered areas from four independent experiments were calculated.

To study the effects of p38 inhibitor (SB203580) and MMP inhibitor NNGH the migration and invasion assays were modified in a way that medium added on top of spheroid or on top of collagen I gel contained DMSO in control (0.05% or 0.38%) or respectively 10μM SB2035 or 1 μM NNGH.

### Cell survival assays

DU145 and PC3 cells were seeded on collagen I (from bovine skin, PureCol^®^, Advanced BioMatrix) or fibronectin (from human plasma, Sigma Aldrich) coated cell culture dishes with KSF medium, allowed to grow for 24 h, then exposed to 50 nM docetaxel (Sigma Aldrich) or for controls with 0.025 % of DMSO in KSF medium for 24h. The surviving cells were collected for flow cytometric analysis.

### Cell proliferation in 2D and 3D cultures

Cell proliferation in 2D monolayer cultures was assayed by estimating the cell numbers using WST-8 Assay (Dojindo Molecular Technologies) according to the manufacturer’s instructions. The cell proliferation in 3D cultures was measured by extraction of genomic DNA from spheroids at 0, 24, 48 and 72 hours with NucleoSpin^®^ Tissue kit (Macherey-Nagel). The concentration of DNA was measured by Qubit™ dsDNA HS Assay Kit (Thermo Fisher Scientific).

To study the effects of p38 inhibitor (SB203580) on proliferation, the media around the agarose micro mold included 0.05 % of DMSO for control or 10μM SB2035.

### Real-time PCR analysis

For mRNA expression analysis cells were grown either as spheroids or on collagen I or fibronectin coated cell culture dishes for 72 hours in serum-free RPMI 1640. cDNA was prepared from 1 μg of RNA with SensiFAST cDNA Synthesis Kit according to the manufacturer’s instructions (Bioline). Primers, designed in house, and probes (Universal ProbeLibrary (Roche)) are listed in Supplementary Table 1. qPCR was performed using a QuantStudio 12K Flex Real-Time PCR System (Thermo Fisher) at the Finnish Functional Genomics Centre (Turku Centre for Biotechnology). PCR reactions were performed in triplicates in a 96-well plate and a 10 μl final reaction volume consisting of 5 μl of ABI TaqMan Universal Master Mix II (Applied Biosystems), 100 nM TaqMan probe, 300 nM of each primer and 2 μl of cDNA sample. The PCR reaction conditions were 95 °C for 10 min, followed by 45 cycles of 95 °C for 15 s and 60 °C for 1 min. Differences in gene expression levels between cell types were calculated using the cycle threshold (C_T_) values, normalized to the C_T_ values of GAPDH and fold changes were calculated based on formula 2^-ΔΔCt^ [51].

### Flow cytometry and cell sorting

For the analysis of α2 integrin surface expression levels, DU145 and PC3 cells were detached with trypsin, washed and subsequently cells were incubated in a blocking buffer (1% FCS in PBS) on ice for 30 min to inhibit unspecific binding of antibodies. Cells were centrifuged and incubated with primary antibody (Supplementary Table 2) for 1 h with gentle agitation at +4°C, washed twice with PBS and incubated for 45 min at +4 °C with 7.5 μg/ml anti-mouse FITC-conjugated secondary antibody (A16167, Life Technologies). For CD44 surface expression level detection cells were treated with FITC-conjugated anti- human CD44 antibody (ab19622, Abcam) for 1h at +4°C. As controls, IgG control or only secondary antibody stained samples were used. Integrin α2 and CD44 cell surface levels were measured immediately after staining procedure with FACSCalibur or Fortessa (BD Biosciences) and results were analyzed with FlowJo program (FlowJo, LLC).

For sorting cells into α2β1^High^ and α2β1^Low^ subpopulations the same staining procedure was performed in the larger scale set up (from 25x10^6^ cells). 20% of cells with lowest and 20% of cells with highest expression levels of α2 integrin were gated and sorted out with FACSaria Ilu Cell Sorter.

The docetaxel treated and normal DU145 cells were analyzed for studying the arresting of cell cycle. Briefly, the cells were seeded as explained in Cell Survival Assay section. Followed by cell staining with propidium iodide (prepared in PBS 1x; 40 mM sodium citrate, 0.3% Triton X-100, and 50 μg/ml propidium iodide) and DNA contents of stained nuclei were analyzed by using LSRFortessa flow cytometer, results were analyzed using the FlowJo program.

### Western blots

Cells were lysed on ice into cold lysis buffer (10 mM Tris-HCl, pH7.4, Triton X-100, supplemented with protease inhibitors 1 mM EDTA, 10 mM NaF, 10μg/ml Aprotin, 10μg/ml Leupeptin, 1 mM Na_3_VO_4_, 2mM PMSF, and 10 mM Na_4_P_2_O_7_). 35 μg of protein in Laemmli SDS-PAGE sample buffer was loaded on gels, and proteins were separated with SDS-PAGE electrophoresis. Proteins were transferred to nitrocellulose membrane, followed by incubation in blocking buffer (5% milk powder, 1% BSA, TBST). The membranes were incubated with primary antibodies (Supplementary Table 2) overnight at +4°C, followed by washing in TBST and incubation with secondary antibody (926-32213, LI-COR). Protein loading was controlled with house-keeping proteins β-actin and Hsp90.

### Colony survival assay

6-well plates were coated either with fibronectin or collagen I (5 μg/cm^2^) for 24 hours at +4°C, and blocked with 1% BSA-PBS for 1 hours at 37°C. DU145 cells were seeded on the coated plates, 200 cells per well. The cells were grown for 10 days in 10% RPMI, after which the medium was changed into KSF medium. On the following day, 50 nM docetaxel or DMSO (as a control) was added to the cells and the colonies were allowed to grow for additional 24 hours. The colonies were fixed with 4% PFA for 15 min, stained with 0.5% crystal violet in 10% ethanol for 15 min, and washed twice with PBS to remove excess stain. The average colony area percentage was calculated using the colony area ImageJ plugin [52].

## SUPPLEMENTARY MATERIALS FIGURES AND TABLES



## Abbreviations

MMP-1: matrix metalloproteinase-1
MAPK: mitogen activated protein kinase
ECM: extracellular matrix
FAK: focal adhesion kinase
KSF: keratinocyte serum-free medium
EGF: epidermal growth factor
CDH5: cadherin 5
SCARA5: scavenger receptor class A member 5
LGI1: leucine rich glioma inactivated 1
KIF26b: kinesin family member 26b
SVEP1: sushi, von Willebrand factor type A, EGF and pentraxin domain containing 1
CHD5: chromodomain-helicase-DNA-binding protein 5
VWA2: von Willebrand factor A domain containing 2
RBP1: retinol binding protein 1
SDC2: syndecan 2
PKP1: plakophilin 1
SYK: spleen associated tyrosine kinase
WT: wild type
