# Supplementary material for: Integrin α2β1 decelerates proliferation, but promotes survival and invasion of prostate cancer cells
Source: Oncotarget. 2018 Aug 21;9(65):32435–47. doi: 10.18632/oncotarget.25945 (PMC6126696; doi:10.18632/oncotarget.25945)
Supplement: Supplementary file 1 [file oncotarget-09-32435-s001.pdf]

Integrin α2β1 decelerates proliferation, but promotes survival and invasion of prostate cancer cells

SUPPLEMENTARY MATERIALS

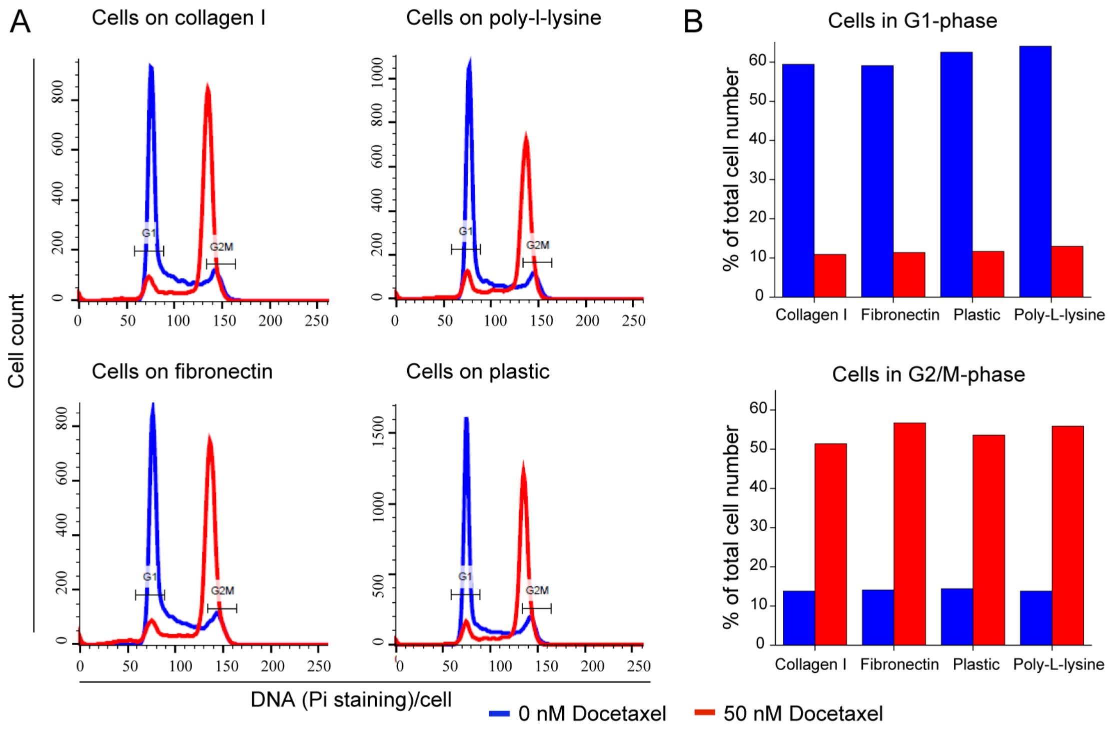

Supplementary Figure 1: Docetaxel treatment arrests DU145 cells into G2/M phase of cell cycle. **(A)** DU145 cells plated on collagen I, fibronectin, poly-l-lysine or plastic were treated with DMSO (blue line) or with 50 nM docetaxel (red line) and the phases of cell cycle were recognized by propidium iodide staining and flow cytometry. **(B)** Graphical presentation of distribution (%) of DU145 cells in G1 and G2/M cell cycle stages in control situation (blue bars) or after the treatment with docetaxel (red bars).

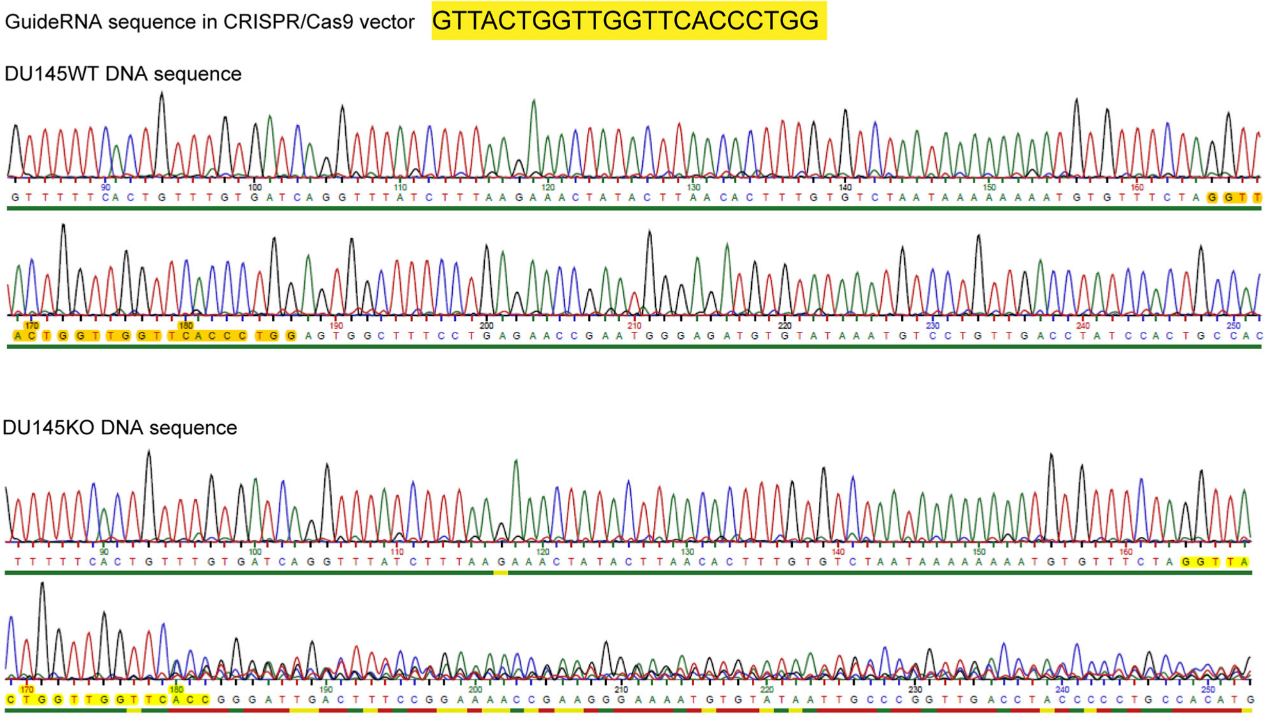

Supplementary Figure 2: DNA Sanger sequencing of control and mutated ITGA2 in DU145 cells. Top chromatogram represents a part of wild-type ITGA2 gene. Guide RNA sequence in Crispr/Cas9 vector is shown in yellow; the entire guide sequence is shown also in DU145WT chromatogram. Bottom chromatogram represents mutated ITGA2 gene DNA sequence from the pool of DU145KO cells with apparent mosaicism after the cutting site.

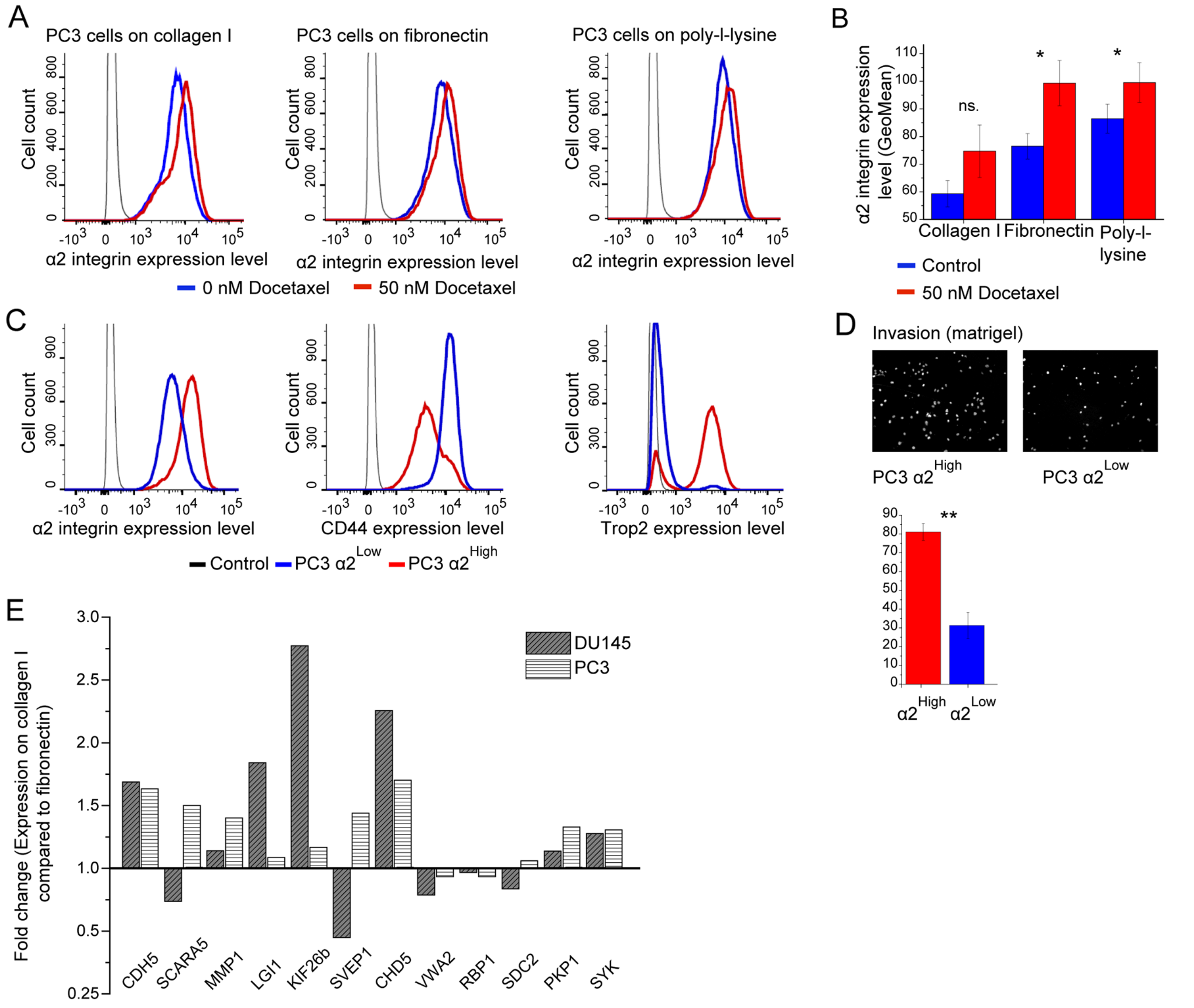

Supplementary Figure 3: Several α2 integrin associated features discovered in DU145cells are characteristic also for PC3 cells. **(A)** The representative FACS plots show increased surface expression levels of α2 integrin on PC3 cells that have been plated on collagen I, fibronectin (5 µg/cm^2) or poly-l-lysine and treated with 50 nM Docetaxel for 24 hours. **(B)** The quantification of α2 integrin cell surface expression based on GeoMean values. Data shown as mean ± SEM (n=3). * = P < 0.05; ns. = not significant. **(C)** PC3 cells were sorted into two sub-populations based on their α2 integrin surface expression levels. Representative FACS plots for α2 integrin, CD44 and Trop2 expression on PC3α2^{High} and PC3α2^{Low} subpopulations analyzed in the 5th passages after sorting. **(D)** Representative microscopic images (10 x) of PC3α2^{High} and PC3α2^{Low} cells that have invaded through matrigel in the transwell invasion assay and the quantification of the invaded cells. Mean ± SEM (n=3). ** = P < 0.01. Student's *t* test. **(E)** Real time PCR analysis of selected, α2β1 integrin associated genes, in DU145WT and in PC3WT cells grown on collagen I or fibronectin for 72 hours. Difference is shown as the fold change of relative mRNA expression level on collagen I compared to expression level on fibronectin. The majority of selected, α2β1 integrin associated genes studied were regulated in the same manner in DU145 and PC3 cells with the exception of tree out of 12 genes.

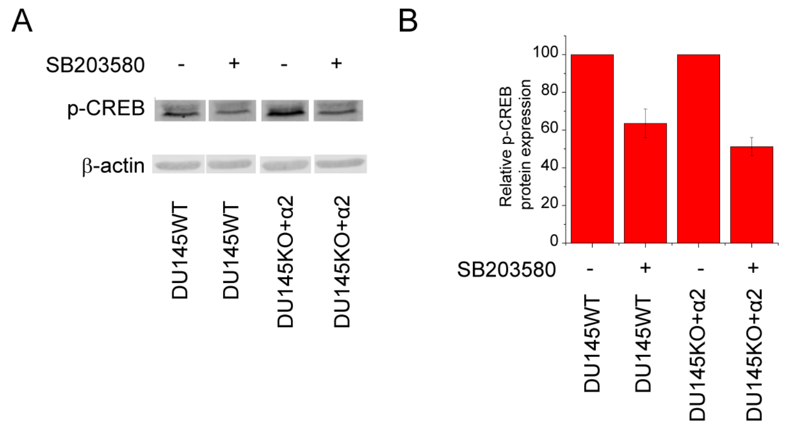

Supplementary Figure 4: SB203580 suppresses phosphorylation of CREB protein, a downstream transcription factor in the p38 MAPK pathway. **(A)** Representative western blot indicating that p38 MAPK inhibitor SB203580 (10μg/ml) successfully decreases activated CREB levels in DU145WT and DU145KO+α2 cells. **(B)** The quantification of the relative amount of phosphorylated CREB. Mean ± SEM (n=3).

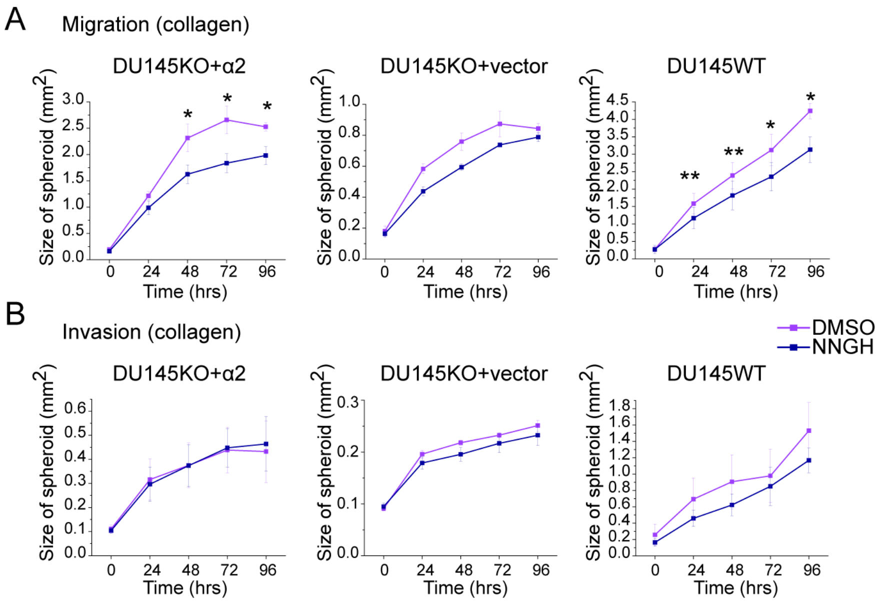

Supplementary Figure 5: MMP inhibitor NNGH suppresses migration of DU145 cells on collagen I. **(A)** Inhibition of MMPs withNNGH (1 μg/ml) results significantly decreased migration of DU145KO+α2 and DU145WT cells on collagen I. The decrease of DU145KO+vector cell migration was not significant. Mean (n = 3) ±SEM. * = P < 0.05, ** = P < 0.01. Student's *t* test. **(B)** Invasion capability of DU145KO+α2, DU145KO+vector or DU145WT was not changed significantly when cells were treated with NNGH (1 μg/ml). Mean (n = 3) ±SEM.

Supplementary Table 1: Primers and probes used for qPCR

Gene	Forward primer	Forward primer	Probe
CHD5	TGGGCTACATGGATGAGAAA	CTCACTCTCCACTCTATCCAAGG	11
CDH5	AAGCCTCTGATTGGCACAGT	GACTCGGAAGAACTGGCCC	58
KIF26B	CGTGTTCTTCACACTGCACAT	CTGCGACCTCCAGACATTC	58
LGI1	TCACTAACCAAACTGACATTCCTAA	ACACGTCCCCTTTCACTGAG	88
PKP1	AAACAGGCACGTCTGGCA	AAGCCATCATACATGGAACCTC	68
RBP1	ACGCTGAGCACTTTTAGGAACT	ATGCCTGTCAGATCCTCCTC	12
SCARA5	TCCAAGCTGAACCTGTGTGA	AGAATCAGGAAGACCAGCAG	56
SDC2	AAACGGACAGAAGTCCTAGCAG	CCTTCATCCTTCTTTCTCATGC	18
SVEP1	TCTCTGTTGGTTTGCCCATA	ATGGAGCCCACAAAAGACTC	26
SYK	AAAGACAAATGGAAAGTTCCTGA	CTTTGTCGATGCGATAGTGC	40
VWA2	GGGTTTGCAGAGGTTGACTG	CTGCGGTACCACCAGGAC	76
MMP1	AAGATGAAACGTGGACCAACAATT	CCAAGAGAATGGAAGAGTTC	*
GAPDH	ACCCACTCCTCCACCTTTGA	TTGCTGTAGCCAAATTCGTTGT	**

* 5'-FAM-CAGAGAGTACAACTTACATCGTGTTGCGGCTC-TAMRA-3'
** 5'-FAM-ACGACCACTTTGTCAAGCTCATTTCCTGGT-TAMRA-3'

Supplementary Table 2: List of primary antibodies used in this study

Target protein	Product code	Company	Dilution
Integrin subunit α2	12F1, BD5555668	BD Pharmingen	7.5 μg/ml
Integrin subunit α2	Mab12332	R&D Systems	1:1000
CD44	Ab19622	Abcam	1:50
β-actin	A1978	Sigma-Aldrich	1:50 000
Hsp90	4877S	Cell Signaling Technology	1:1000
Phospho-p38	9215L	Cell Signaling Technology	1:1000
Phospho-FAK	3283S	Cell Signaling Technology	1:1000
Phospho-ERK	5726S	Cell Signaling Technology	1:1000
Phospho-CREB	MAB6906	BD Biosciences	0.5 μg/ml
FAK	610088	BD Biosciences	1:1000
ERK	9102S	Cell Signaling Technology	1:1000
Trop2	MAB650	BD Biosciences	1:500
